# Beyond Abandonment to Next Steps: Understanding and Designing for Life after Personal Informatics Tool Use

**DOI:** 10.1145/2858036.2858045

**Published:** 2016-05

**Authors:** Daniel A. Epstein, Monica Caraway, Chuck Johnston, An Ping, James Fogarty, Sean A. Munson

**Affiliations:** 1 Computer Science & Engineering, DUB Group, University of Washington; 2 Human Centered Design & Engineering, DUB Group, University of Washington

**Keywords:** Personal informatics, self-tracking, abandonment, H.5.m. Information interfaces and presentation (e.g.,HCI)

## Abstract

Recent research examines how and why people abandon self-tracking tools. We extend this work with new insights drawn from people reflecting on their experiences after they stop tracking, examining how designs continue to influence people even after abandonment. We further contrast prior work considering abandonment of health and wellness tracking tools with an exploration of why people abandon financial and location tracking tools, and we connect our findings to models of personal informatics. Surveying 193 people and interviewing 12 people, we identify six reasons why people stop tracking and five perspectives on life after tracking. We discuss these results and opportunities for design to consider life after self-tracking.

## Introduction

Personal informatics is defined as the process of collecting and reflecting on personal information [12], and is now a common practice in the lives of many people [7]. However, people over time come to temporarily lapse or permanently discontinue self-tracking [4,5,6,11]. We study abandonment of self-tracking tools to gain insight into how to design tools that: (1) better align with tracking objectives and practices, and (2) support better abandonment experiences.

This paper extends current understanding of abandonment with insights drawn from people reflecting on their experiences after they stopped self-tracking. As part of this, we examine how designs can continue to influence people even after abandonment. We extend recent work examining self-tracking technology abandonment in health and wellness [4,5,11] by contrasting it with abandonment in other self-tracking domains, specifically finance and location. We frame these findings in models of how people use personal informatics tools [6,12], and we identify and discuss how self-tracking barriers lead to abandonment.

We survey 193 people who formerly tracked their physical activity, finances, or location, conduct 12 interviews, and distill themes from this qualitative data. We extend prior work by identifying six reasons people stop tracking and five perspectives on life after tracking among the three studied domains. Our results contribute to a growing understanding of self-tracking abandonment, and surface opportunities for design to consider life after tracking.

## Background and Related Work

To characterize how people use self-tracking tools, Li et al. introduce a five-stage model of personal informatics, which emphasizes barriers to tracking toward a goal of reflection and presumed action [12]. This model has been modified and expanded, noting people can reflect on data [3] and ultimately change habits [16] in the midst of tracking. Epstein et al. characterize challenges in lived informatics [14], developing a model of tool use in everyday life that surfaces lapsing and stopping as major components [6].

Avoiding or discontinuing the use of technology is common practice. Baumer et al. enumerate motivations for not using Facebook, including concerns for data use and privacy as well as avoiding addiction [2]. In the domains of physical activity and health and wellness more broadly, recent work has explored reasons people stop using self-tracking tools. Schwanda et al. interview people using Wii Fit, finding they begin other exercise activities and abandon the technology, regarding the abandonment as a success [15]. Clawson et al. extend this “happy abandonment,” suggesting designs should support people who no longer feel the need to track [4]. Tools sometimes satisfy people's curiosity about their habits, rendering tracking no longer important [6,11]. People find tools frustrating or time-consuming, ultimately not worth the time investment [5,11]. We extend and contrast such findings in the domains of finance and location, building a broader understanding of abandoning tracking.

## Data Collection and Analysis

We conducted a series of surveys using Amazon Mechanical Turk with people from the United States who had completed at least 1,000 HITs with a 95% acceptance rate. To identify people who had previously tracked, participants completed a 2-minute screener survey ($0.50 compensation). Of 640 completed surveys, we identified 133, 101, and 91 people who had tracked their physical activity, finances, and location (271 unique people). We then conducted two follow-up surveys (screened participants could take either). The first examined self-tracking abandonment practices, requiring about five minutes ($1.00 compensation). The second emphasized life after tracking, requiring about 10 minutes ($2.00 compensation). Of people who tracked physical activity, finances, and location, 64, 61, and 50 people completed the first follow-up survey, and 68, 56, and 42 people completed the second. 193 unique people (aged 18-63, mean 31.6) completed at least one survey, including 109 male, 82 female, and 2 people who declined to report gender. Length of tool use varied substantially, ranging from less than a week to over two years. 49% had used more than one tracking tool (average 1.7 tools used). Additional details and survey materials are in the online appendix.

Based on responses to the first survey, we invited 34 respondents to participate in an interview, targeting people with either particularly representative or unique experiences, yielding 6 interviewees. We supplemented these with 6 interviewees recruited through a convenience sample (university mailing lists and social network posts), for a total of 12 (4 male, 8 female). Interviews lasted about an hour, and interviewees were compensated $20.

Four researchers used open coding to identify themes in responses and transcripts. Two then coded independently, discussing and refining codes to reach high agreement (Cohen's Kappa 0.74 to 1.0 for 11 codes). We quote survey participants with pXX, and interview participants with iXX.

## Why People Stop Tracking

Participants in our study, especially those who had tracked for health and wellness, stopped using their tools for many of the reasons identified in prior research. We also found reasons not previously identified, especially among trackers in other domains. We ground our discussion of why people stop tracking in models of personal informatics [12] and lived informatics [6], and contrast our findings with prior work. These categories do not cover all the reasons people stop tracking (e.g., tools break, people switch phones [14]), but capture the most prominent themes we found in our data.

### Cost of collecting and integrating

The cost of tracking often leads to abandonment, consistent with prior health and wellness results [4,5,11]. People find tracking “*a hassle,*” feel “*lazy,*” or “*lose interest*” and stop tracking (20, 16, and 6 people). The habit of collecting data can often be difficult to maintain in tools that require regular manual entry, such as financial spreadsheets or food journals [5,6]: “*I got behind on keeping up with it and couldn't find the time to start back up*” (p112, 18 others). Others find tracking too tiring “*I got burnt out on [keeping a financial spreadsheet]*” (p14).

### Cost of having or sharing the data

45.2% of respondents who stopped tracking location did so due to concerns for data sharing. This surfaces within Li et al.'s model as part of *integration* between the location data collected and the social context in which it is shared [12]. Although concerns with regard to privacy in location tracking and sharing are well-documented [1,13], we note a connection between sharing concerns and abandonment of location self-tracking. People were concerned about what friends could see “*I don't want people knowing where I am all the time*” (p161, 11 others) as well as companies using information about them “*I don't want a record of my activities being reviewed or sold to try to sell me better ads*” (p61, 6 others). Although these concerns were largely expressed by people tracking location, emerging new domains (e.g., biometric data such as heart rate or blood pressure) may present similar concerns, especially as more personal informatics tools rely on cloud storage or processing.

### Discomfort with information revealed

During *reflection*, tracking can highlight perceived shortcomings in behavior or behavior change, and people can find this uncomfortable. p133 “*didn't like being so aware of how little money I had,*” while p178 stopped tracking because she “*had to witness my weight fluctuations every day.*” For p163, entering new expenses was unpleasant: “*I felt guilty every time I tracked an expense that was not a necessity.*” Prior work has found food journals can feel judgmental when people exceed their calorie budgets [5]. We extend this finding, noting that tools in other domains can create feelings of judgment.

Abandoning tracking may be the easiest way to address this discomfort. p23 stopped tracking because she “*felt discouraged with my lack of progress*” and p3 “*didn't feel like I was getting the results that I wanted.*” Tracking emphasized the difficulty of p144's circumstances and made her feel even worse about her situation: “*the decision to stop tracking made me very sad, like it was the final thing in admitting how bad my [financial] situation had become.*” When people do not feel tools help them *act* to change the situation, tracking may only add to their frustration.

### Data quality concerns

People often desire greater accuracy than their tools provide [12]: “*the calories burnt seemed so random, and didn't line up with other online sources*” (p123, 19 others), or find data unreliable “*the GPS would lose my location and stop tracking my run*” (p139), leading to imperfect personal data. This problem in *collection* inhibits effective *reflection* and consequently *action*.

### Learned enough

People often begin tracking to learn their habits, with a goal of *action* [6,12]. Moreover, Rooksby et al. find people track over short-term periods in support of long-term goals [14]. We also found this practice in our participants. Six people felt no reason to continue tracking after learning what they needed in order to act: “*I was very familiar with the different routes I was taking when I was running, so I eventually just sort of phased out MapMyRun. It wasn't beneficial to me anymore*” (p20). After people develop a skill, the potential benefits of tracking change and it can become unnecessary. p29 “*was able to figure my distance and calories burned without [MapMyRun]*” and p89 “*was able to keep track [of my finances] in my head.*”

People also track to develop and adhere to plans, such as exercise, weight loss, or budgets [6,8,9,10]. Although helpful in developing a plan, some people find that “*once I had that plan, the Fitbit was no longer necessary*” (p171, 5 others). p146 “*felt that I could take charge of keeping my own lifestyle*” and thought tracking was no longer required.

### Life circumstances change

As noted in the lived informatics model, lives and routines may change, leading people to intentionally or unintentionally suspend tracking [6]. People regularly change their activities to ones their existing tracking tools do not support, such as p115 who “*gave up running and started to do other types of exercise.*” p127 “*moved to an area that did not have a good walking trail*” and “*had to switch to indoor activity and MapMyRun isn't great for that.*” Other life events may force people to give up the activity they tracked, such as for p17 “*I became pregnant and was not able to exercise anymore because my pregnancy was high risk,*” p181 “*I stopped riding my bike due to a serious injury,*” and 8 others. Other life events often take priority over keeping up with tracking, such as for p133, who used to track finances and then “*got divorced and had other things to focus on.*”

Some people described stopping tracking finances when they did not have any money to track. For i1, “*it came to a point where as soon as I had money it had to immediately go to pay for something that was needed, so there really wasn't anything to keep track of*.” These were often caused by unemployment “*I lost my job and my income dropped*” (p109, 5 others) or career changes “*I went to grad school and thus had no income*” (p82, 5 others). However, stopping tracking can also signal an improving financial situation, such as for p50 “*I was out of debt and making good money*” (2 others).

## Life After Tracking

People had varied opinions and feelings about abandoning tracking. Table 1 shows the relationship between reasons for abandonment and perspectives on life after tracking reported by participants in our second survey. Percentages correspond to agreement between the two coders.

### No major effect

Interest in tracking often faded without leaving a strong impression, especially for people who felt the cost of tracking was too high to continue. People described this perspective across all three of the tracking domains we surveyed. i7 used MapMyRun, but “*I didn't think about it until your survey, that actually was when I remembered, ‘Yeah, I did do that.’*” Some people, such as p55 and 5 others, started to track out of curiosity but were not particularly invested in the activity: “*I felt indifferent about [tracking] because I wasn't that into trying to increase my physical activity.*” Like p55, most who started out of curiosity and later abandoned tracking then feel indifferent about their experience. p110 stopped tracking his location because he “*simply forgot to do so,*” and he had few thoughts about his tracking experiences: “*I don't feel bad. I don't feel much about it. It just kind of happened.*”

### Frustration

Tracking tools often do not match people's expectations [4]. After abandonment, some people remain frustrated with their lack of success tracking and attribute that frustration differently. Some felt frustrated with the cost of tracking [11], as witsh p57 who stopped using a Fitbit: “*I feel more frustrated than anything… I want to track my data. I just would rather it be effortless.*” Others felt frustrated that a situation outside their control prevented tracking: “*it was frustrating to know that I needed to make time to do the tracking, but the needs of my family outweighed my own personal goals*” (p23 and 4 others). As tool design evolves and lives change, people may decide to return. Similar to people interviewed by Lazar et al. [11], 16 people planned to return, including p19: “*I'm ok with my decision to stop, but I do think that I will probably do it again in the future*.”

Frustration with the effectiveness of tracking was uncommon among financial and location trackers in our study. This may be because location trackers typically start tracking out of curiosity rather than a desire to change habits [6], while financial trackers often want a *financial touch* (e.g., awareness of current status) [10]. Because they did not initially anticipate action, they may be less frustrated by abandonment.

### Guilt

Some people feel guilty for not making tracking a habit, though they found the cost of tracking too high to continue. This was also more common among people who started tracking with the intent of changing physical activity (16.2% of people who tracked activity) or financial habits (8.9% of people who tracked finances). i11 tried to use a spreadsheet to track her finances, stopped, and eventually “*started feeling guilty… I shouldn't have stopped finance tracking.*” She eventually tried to use Mint, but stopped again. She still feels guilty about stopping: “*I really should get back to using this or something similar again… I ought to be doing something like this. So yes, guilt.*”

People often blame themselves for not continuing to track, particularly in physical activity. p87 stated, “*I feel like I am wasting my potential by not keeping on top of tracking,*” while p116 felt “*regret because I could have seen my progress or lack thereof over time.*” p188 stopped tracking his activity because “*at the time I thought I could handle it on my own.*” He was sadly unsuccessful: “*I regret it deeply. My workouts have suffered if I do them and most weeks I do not.*” A few felt guilty for abandoning tracking after a change in life circumstances (5.9% and 5.4% of people no longer tracking physical activity or finances): p97 got sick, stopped tracking her activity, and now feels “*ashamed that I haven't restarted, because I was doing so well*.”

### Freedom

Self-tracking can be time-consuming, difficult, and “*more of a hassle than a pleasure*” (p65). Some are glad to be done, even if they were not successful. For p52, financial tracking “*felt more and more cumbersome … it used up a fair bit of my time*.” Ceasing tracking “*felt like a burden was lifted off of me. Although I wanted to continue tracking, I felt this was the right decision… I wanted some of my free time back.*” A few people who stopped after they learned enough felt free from needing to track. After i10 felt she understood her spending, she “*was able to quit.*” p163 avoided the guilt tracking caused and still successfully kept her budget: “*I feel that I am still able to stay within my budget while no longer feeling guilty for any of my purchases that are not necessities.*”

Location trackers most commonly felt free from tracking their data, particularly those who had concerns about having or sharing data (33.3% of people surveyed). p18 felt free from friends knowing, “*I feel like I can move around freely without everyone knowing my every move,*” while p25 appreciated freedom from businesses tracking him: “*I didn't like thinking that outside agencies could see my location tracking and it made me feel very exposed.*” p188 found tracking his location encouraged bad habits: “*I NEEDED to be the mayor of places that were important to me so I spent more time there than I needed to and spent more money… it had turned into a stress rather than something fun*.” Abandoning freed him from the added stress of competition.

### Continued use of knowledge or skills

Many people internalize habits developed when tracking and continue to apply their knowledge after abandonment. Financial trackers such as p45 and 11 others learned their spending habits and no longer needed to track: “*I knew that I could spend wisely… my finances are still good, and I haven't had any issues because I've stopped tracking.*” p135 stopped tracking her physical activity because she “*had a very good idea of the amount of steps I was taking without the app… I knew without the app if I was accomplishing my goal.*”

Tracking helps others make structural changes to their lives. p118 “*set a budget that I now carefully follow*,” and felt “*the financial tracking software I used are like training wheels. I used the training wheels to develop balance. It was time for the training wheels to come off*.” p89 changed her habits and felt tracking was no longer important: “*I made changes to my spending and did not need to keep close tabs… I have kept up with my changes and am still doing well*.” p40 started tracking because he “*had a goal of being able to run a 5K without stopping*.” He “*felt tracking did its job*” and stopped, but is “*still running 5K's when I can*.”

People successfully use tracking to change habits, and some feel that continuing to track is unnecessary to sustain the change. 7.1% of respondents no longer tracking their finances reported using knowledge they gained from tracking, despite stopping tracking due to data collection barriers. This suggests *reflection* and *action* can occur long after people abandon data *collection*. Abandonment is thus not always indicative of failure nor the successful end of a process, but could rather be a sign of diminishing returns or a redefinition of goals.

## Discussion and Conclusion

Self-tracking is not an effective or beneficial experience for everyone. Many recall their tracking experience with indifference, as just another piece of technology that did not fit in their lives. Others feel frustration that tracking did not support their goals and blame either themselves or the tools. Still others feel guilty for abandoning tracking and wish they had found the will or time to resume. Designers should explore how to appropriately facilitate reengagement and how designs can support a more successful experience in returning. i8 stopped using Mint five years ago, yet “*I still get emails from [Mint] that I ignore… I don't care about them.*” Perhaps tracking tools can promote programs tailored to resuming tracking (e.g., restarting physical activity), rather than nagging notifications pointing out a lack of tracking.

Models of personal informatics in everyday life further include the process of *deciding* to track and *selecting* a tool [6,12]. Our participants had all tried tracking, but we believe many people consider tracking and abandon the idea before ever starting. Future work should study why people abandon tracking before they start, which may inform better design for decisions about whether to track and which tool to use.

We contribute an understanding of life after tracking. Having tracked has no lasting effect for some people, while others continue to apply the lessons they learned about their habits or how to act. For others, tracking was a frustrating experience they wished fit better into their lives. Some felt guilty about stopping, while others felt free from the burdensome or invasive activity of tracking. We surface open opportunities to design for life after tracking, promoting happy abandonment, and designing tools to best support people who want to return to tracking.

## Supplementary Material

Appendix: First follow up survey

Appendix: Participant demographics

Appendix: Screener survey

Appendix: Second follow up survey

Description of appendices

## Figures and Tables

**Table 1:** Participant perspectives on life after tracking grouped by reason for abandonment. The first row shows the cumulative percentage of each reason for why people stop tracking

	Reason for abandoning tracking					
	Cost of collecting and integrating	Cost of having or sharing the data	Discomfort with the information revealed	Data quality concerns	Learned enough	Life circumstances change
**Activity**	**45.6%**	**2.9%**	**5.9%**	**5.9%**	**11.8%**	**13.2%**
No effect	22.1%	1.5%	1.5%	2.9%	4.4%	1.5%
Frustration	10.3%	0%	1.5%	1.5%	0%	0%
Guilt	16.2%	0%	1.5%	1.5%	1.5%	5.9%
Freedom	2.9%	0%	1.5%	0%	0%	2.9%
Use skills	1.5%	0%	0%	1.5%	8.8%	1.5%
**Location**	**42.9%**	**45.2%**	**0%**	**9.5%**	**0%**	**2.4%**
No effect	21.4%	7.1%	0%	4.8%	0%	2.4%
Frustration	0%	0%	0%	0%	0%	0%
Guilt	0%	0%	0%	0%	0%	0%
Freedom	16.7%	33.3%	0%	4.8%	0%	0%
Use skills	0%	0%	0%	0%	0%	0%
**Finances**	**57.1%**	**1.8%**	**5.4%**	**0%**	**14.3%**	**10.7%**
No effect	25.0%	0%	0%	0%	0%	3.6%
Frustration	5.4%	0%	0%	0%	0%	1.8%
Guilt	8.9%	0%	1.8%	0%	0%	5.4%
Freedom	17.9%	1.8%	3.6%	0%	1.8%	3.6%
Use skills	7.1%	0%	0%	0%	14.3%	0%
