# Supplementary material for: Beyond Abandonment to Next Steps: Understanding and Designing for Life after Personal Informatics Tool Use
Source: Proc SIGCHI Conf Hum Factor Comput Syst. Author manuscript; Available in PMC 2017 May 12. (PMC5428074; doi:10.1145/2858036.2858045)
Supplement: Appendix: First follow up survey [file NIHMS855559-supplement-Appendix__First_follow_up_survey.pdf]

Physical activity follow-up

Thank you for taking part in our follow-up survey! This survey only has one page, and you will receive a code to paste into Mechanical Turk upon its completion.

1) Which of the following tools did you use to track your physical activity?*

[ ] Fitbit

[ ] Jawbone Up

[ ] Nike Fuel Band

[ ] MapMyRun

[ ] RunKeeper

[ ] Apple HealthKit

[ ] Myfitnesspal

[ ] Google fit

[ ] Other wearable devices: ____

[ ] Other phone apps: ____

2) In total, how long did you track your physical activity for?*

( ) Less than one week

( ) 1 week - 1 month

( ) 1-3 months

( ) 3-6 months

( ) 6 months - 1 year

( ) 1-2 years

( ) more than 2 years

( ) I don't remember

3) How long ago did you stop tracking?*

( ) Less than one week ago

( ) 1 week - 1 month ago

( ) 1-3 months ago

( ) 3-6 months ago

( ) 6 months - 1 year ago

( ) 1-2 years ago

( ) more than 2 years ago

( ) I don't remember

4) Which of the following is most true for you?*

( ) Before I started tracking, I did not know if I wanted to change my behavior.

( ) Before I started tracking, I wanted to maintain my current behavior or I did not want to change my behavior.

( ) Before I started tracking, I wanted to change my behavior and did not have a plan for how to do so.

( ) Before I started tracking, I wanted to change my behavior and had a plan for how to do so.

5) Why did you stop tracking?*

____

____

____

____

6) What about your tracking experience did you find *frustrating*?*

____

____

____

____

7) At the time you decided to stop tracking, how did you feel about this decision? Why?*

____

____

____

____

8) How do you feel *now* about your decision to stop tracking? Why?*

____

____

____

____

9) Do you ever think about trying to track again? What would or would not make you want to return to tracking?*

____

____

____

____

10) How do you think your habits have changed since you stopped tracking? Do you think you are more, less, or about as physically active?*

____

____

____

____

11) How old are you?

____

12) What gender do you identify as?

____

Thank You!

Thank you for taking our survey! Please write down or copy-and-paste the following code into Amazon Mechanical Turk:

[survey("session id")]
