# Supplementary material for: Beyond Abandonment to Next Steps: Understanding and Designing for Life after Personal Informatics Tool Use
Source: Proc SIGCHI Conf Hum Factor Comput Syst. Author manuscript; Available in PMC 2017 May 12. (PMC5428074; doi:10.1145/2858036.2858045)
Supplement: Appendix: Participant demographics [file NIHMS855559-supplement-Appendix__Participant_demographics.pdf]

Physical Activity (91 people surveyed)

Most common tools used:

- MyFitnessPal (45 people)
- MapMyRun (23 people)
- Fitbit (18 people)
- RunKeeper (16 people)
- Nike+ (11 people)
- Apple HealthKit (5 people)
- Nike Fuel Band (4 people)
- Google Fit (4 people)
- Footsteps (4 people)

Time Tracked:

- Less than 1 week: 7 people
- 1 week – 1 month: 17 people
- 1 month – 3 months: 23 people
- 3 months – 6 months: 12 people
- 6 months – 1 year: 11 people
- 1 year – 2 years: 5 people
- More than 2 years: 2 people
- I don’t remember: 4 people

Location (65 surveyed)

Most common tools used:

- Location tagging on Facebook (52 people)
- Foursquare or Swarm (29 people)
- Location tagging on Instagram (20 people)
- Google Latitude (18 people)
- Location tagging on Twitter (14 people)
- FindMyFriends (4 people)

Time Tracked:

- Less than 1 week: 13 people
- 1 week – 1 month: 14 people
- 1 month – 3 months: 6 people
- 3 months – 6 months: 2 people
- 6 months – 1 year: 7 people
- 1 year – 2 years: 6 people
- More than 2 years: 1 person
- I don’t remember: 12 people

Finances (74 surveyed)

Most common tools used:

- Spreadsheets (49 people)
- Mint (36 people)
- Apps provided by banks (32 people)
- Quicken (13 people)
- You Need A Budget (8 people)

Time Tracked:

- Less than 1 week: 5 people
- 1 week – 1 month: 9 people
- 1 month – 3 months: 16 people
- 3 months – 6 months: 15 people
- 6 months – 1 year: 8 people
- 1 year – 2 years: 8 people
- More than 2 years: 6 people
- I don’t remember: 4 people
