# Supplementary material for: Beyond Abandonment to Next Steps: Understanding and Designing for Life after Personal Informatics Tool Use
Source: Proc SIGCHI Conf Hum Factor Comput Syst. Author manuscript; Available in PMC 2017 May 12. (PMC5428074; doi:10.1145/2858036.2858045)
Supplement: Appendix: Screener survey [file NIHMS855559-supplement-Appendix__Screener_survey.pdf]

Tried Tracking Screener

Instructions

In this survey, we're interested in gathering opinions from people who have tried applications or devices to track physical activity, location, or finances. We will begin with a few short screener questions. If you fit the criteria for the survey, we will contact you with a link to the full version.

You will be compensated $0.50 for completing the screener questions, and they should take no more than 3 minutes to complete. We will compensate $1.00 each of the full surveys (which should take no more than 8 minutes to fill out), should you qualify for them.

Physical activity tracking

Many devices and mobile apps are geared toward tracking physical activity. Examples of devices that track physical activity are Fitbit, Jawbowne Up, and the Nike Fuel Band. Examples of mobile apps that track physical activity are RunKeeper, Strava, MyFitnessPal, and MapMyRun.

1) Have you ever tried technology to track your physical activity as defined above?*

( ) Yes

( ) No

2) What device(s) and app(s) have you tried? Please list all that come to mind.*

____

Experience tracking physical activity

3) When was the last time you used a tool to track your physical activity?*

( ) Earlier today

( ) Yesterday

( ) Within the past week

( ) Within the past month

( ) Within the past 3 months

( ) Within the past 6 months

( ) Within the past year

( ) More than one year ago

( ) I don't remember

4) Do you consider yourself someone who *currently* uses tools to track their physical activity?*

( ) Yes

( ) No

5) Have you ever temporarily stopped tracking your physical activity? If so, why?*

____

____

____

____

6) Why have you not used a physical activity tracking tool or reviewed your data in the past month?*

____

____

7) Why did you stop tracking your physical activity?*

Finance tracking

For the context of this survey, we define tracking your finances as inclusive of, but not limited to: reviewing transaction statements, checking balances, and managing discretionary spending. Some digital tools for tracking finances include Mint.com, Quicken, or using a spreadsheet such as Excel or Google Docs.

8) Have you ever tried tracking your finances with a digital tool as defined above?*

( ) Yes

( ) No

9) What app(s) and tool(s) have you tried? Please list all that come to mind.*

____

Experience tracking finances

10) When was the last time you used a tool to track your finances?*

( ) Earlier today

( ) Yesterday

( ) Within the past week

( ) Within the past month

( ) Within the past 3 months

( ) Within the past 6 months

( ) Within the past year

( ) More than one year ago

( ) I don't remember

11) Do you consider yourself someone who *currently* uses tools to track their finances?*

( ) Yes

( ) No

12) Have you ever temporarily stopped tracking your finances? If so, why?*

____
____
____
____

13) Why have you not used a financial tracking tool or reviewed your data in the past month?*

____
____
____
____

14) Why did you stop tracking your finances?*

____
____
____
____

Location tracking

There are many mobile applications that use your GPS location to control features and personalize feedback. There are apps that highlight location tracking as a key feature (Foursquare, Moves, etc.) and there are apps that allow you to tag your location, but location tracking is not the main purpose (Facebook, Twitter, Instagram, etc.). For the purposes of this survey, disregard apps that access your location, but you cannot use to look back at your location history (GoogleMaps, Waze).

15) Have you ever tried to track your location with an application as defined above?*

( ) Yes

( ) No

16) What app(s) and tool(s) have you tried? Please list all that come to mind.*

____

Experience tracking location

17) When was the last time you used a tool to track your location?*

( ) Earlier today

( ) Yesterday

( ) Within the past week

( ) Within the past month

( ) Within the past 3 months

( ) Within the past 6 months

( ) Within the past year

( ) More than one year ago

( ) I don't remember

18) Do you consider yourself someone who *currently* uses tools to track their location?*

( ) Yes

( ) No

19) Have you ever temporarily stopped tracking your location? If so, why?*

____

____

____

____

20) Why have you not used a location tracking tool or reviewed your data in the past month?*

____

____

____

____

21) Why did you stop tracking your location?*

____

____

____

____

Interest in tracking

22) Have you ever considered buying a wearable self-tracker, such as a FitBit, Jawbone UP, Microsoft Band, or Misfit Shine? If so, what stopped you from purchasing them?*

____

____

____

____

23) Consider the hypothetical scenario: you win a wearable self-tracking device in a raffle. Would you use it? Why or why not?*

____

____

____

____

Thank You!

Thank you for taking our survey! Please write down or copy-and-paste the following code into Amazon Mechanical Turk:

[survey("session id")]
