# Supplementary material for: Beyond Abandonment to Next Steps: Understanding and Designing for Life after Personal Informatics Tool Use
Source: Proc SIGCHI Conf Hum Factor Comput Syst. Author manuscript; Available in PMC 2017 May 12. (PMC5428074; doi:10.1145/2858036.2858045)
Supplement: Appendix: Second follow up survey [file NIHMS855559-supplement-Appendix__Second_follow_up_survey.pdf]

Tried Tracking Physical Activity

Instructions

In this survey, we're interested in gathering opinions from people who tried applications or devices to track physical activity and no longer use them. You will be compensated $1.00 for completing this survey, which should take no more than 8 minutes to fill out.

Physical activity tracking

Many devices and mobile apps are geared toward tracking physical activity. Examples of devices that track physical activity are Fitbit, Jawbowne Up, and the Nike Fuel Band. Examples of mobile apps that track physical activity are RunKeeper, Strava, MyFitnessPal, and MapMyRun.

1) Have you ever tried technology to track your physical activity as defined above?*

( ) Yes

( ) No

2) Why did you decide to start tracking your physical activity?*

____

____

____

____

3) What wearable device or phone app have you used most recently?*

( ) Fitbit

( ) Jawbone Up

( ) Nike Fuel Band

( ) Map My Run

( ) RunKeeper

( ) Apple HealthKit

( ) Myfitnesspal

( ) Google fit

( ) Other wearable device: ____

( ) Other phone app: ____

4) Have you tried any other wearable devices or phone apps? Which other ones have you tried?

[ ] Fitbit

[ ] Jawbone Up

[ ] Nike Fuel Band

[ ] Map My Run

[ ] RunKeeper

[ ] Apple HealthKit

[ ] Myfitnesspal

[ ] Google fit

[ ] Other wearable device: ____

[ ] Other wearable device: ____

[ ] Other wearable device: ____

[ ] Other phone app: ____

[ ] Other phone app: ____

[ ] Other phone app: ____

Physical activity frecuency

5) For how long did you use [page("piped title")]?

( ) Less than 1 week

( ) 1 week - 1 month

( ) 1-3 months

( ) 3-6 months

( ) 6 months - 1 year

( ) 1-2 years

( ) More than 2 years

( ) I don't remember

6) Why did you stop using or switch away from [page("piped title")] to track your physical activity?*

____

____

____

____

Experience tracking physical activity

7) For how long did you use [question("title"), id="21"]?*

( ) Less than 1 week

( ) 1 week - 1 month

( ) 1-3 months

( ) 3-6 months

( ) 6 months - 1 year

( ) 1-2 years

( ) More than 2 years

( ) I don't remember

8) Why did you stop using [question("title"), id="21"] to track your physical activity?*

____

____

____

____

9) Please rate your agreement with the following statements:*

	**Strongly disagree**	**Disagree**	**Neutral**	**Agree**	**Strongly agree**	**Not Applicable**
I am concerned my physical activity data is too personal to share with a physical activity	( )	( )	( )	( )	( )	( )

tracking application.						
I am concerned a physical activity tracking application might not keep my physical activity data secure.	( )	( )	( )	( )	( )	( )

Demographics

The demographic questions on this page are voluntary, and will not be used in conjunction with your results.

10) How old are you?

____

11) What gender do you identify as?

( ) Male

( ) Female

( ) Other: ____

12) What is your ethnicity?

( ) American Indian or Alaskan native

( ) Asian or Pacific Islander

( ) Hispanic/Latino

( ) Black/African American

( ) White/Caucasian

( ) Other: ____

13) What state do you live in?

( ) Alabama

( ) Alaska

( ) American Samoa

( ) Arizona

( ) Arkansas

( ) California

( ) Colorado

( ) Connecticut

( ) Delaware

( ) District of Columbia

( ) Federated States of Micronesia

( ) Florida

( ) Georgia

( ) Guam

( ) Hawaii

( ) Idaho

( ) Illinois

( ) Indiana

( ) Iowa

( ) Kansas

( ) Kentucky

( ) Louisiana

( ) Maine

( ) Marshall Islands

( ) Maryland

( ) Massachusetts

( ) Michigan

( ) Minnesota

( ) Mississippi

( ) Missouri

( ) Montana

( ) Nebraska

( ) Nevada

( ) New Hampshire

( ) New Jersey

( ) New Mexico

( ) New York

( ) North Carolina

( ) North Dakota

( ) Northern Mariana Islands

( ) Ohio

( ) Oklahoma

( ) Oregon

( ) Palau

( ) Pennsylvania

( ) Puerto Rico

( ) Rhode Island

( ) South Carolina

( ) South Dakota

( ) Tennessee

( ) Texas

( ) Utah

( ) Vermont

( ) Virgin Islands

( ) Virginia

( ) Washington

( ) West Virginia

( ) Wisconsin

( ) Wyoming

14) How would you describe where you live?

( ) Urban

( ) Suburban

( ) Rural

15) What is the highest degree or level of school you have completed? *If currently enrolled, highest degree received*.

( ) Less than high school

( ) High school graduate or equivalent (e.g. GED)

( ) Some college, no degree

( ) Associate's degree

( ) Bachelor's degree

( ) Graduate or professional degree (e.g. PhD, MD, JD)

( ) Other: ____

16) What is your occupation?

____

Interview

17) We are interested in further interviewing people about their experiences. If you are selected, the interview would last up to one hour, and we will compensate with an additional $20 Amazon gift card. Would you be interested in participating in such an interview?

( ) Yes

( ) No

18) Please enter the email address we should use to contact you for an interview.

____

Thank You!

Thank you for taking our survey! Please write down or copy-and-paste the following code into Amazon Mechanical Turk:

[survey("session id")]
