# Supplementary material for: Beyond Abandonment to Next Steps: Understanding and Designing for Life after Personal Informatics Tool Use
Source: Proc SIGCHI Conf Hum Factor Comput Syst. Author manuscript; Available in PMC 2017 May 12. (PMC5428074; doi:10.1145/2858036.2858045)
Supplement: Description of appendices [file NIHMS855559-supplement-Description_of_appendices.pdf]

Readme

There are four files in our supplementary materials.

- Questions used in the screener survey
- Questions used in the first follow-up survey for they physical activity domain
- Questions used in the second follow-up survey for they physical activity domain
- Participant demographic information from our two follow-up surveys

The questions used in the follow-up surveys were identical in the location and finance domains, swapping out specific wording as appropriate.

All files are PDFs and can be opened with a standard PDF reader (e.g. Adobe PDF, Preview).
